# No safe place for war survivors: War memory, event exposure, and migrants' psychological trauma

**DOI:** 10.3389/fpsyt.2022.966556

**Published:** 2023-01-11

**Authors:** Muhammad Sohail Ahmad, Zainab Bukhari, Sundas Khan, Imran Ashraf, Asma Kanwal

**Affiliations:** ^1^Division of Arts and Social Sciences, Department of English, University of Education, Lahore, Pakistan; ^2^Department of Information and Communication Engineering, Yeungnam University, Gyeongsan, South Korea

**Keywords:** war trauma, double wounds, loss, narration, trauma as healer

## Abstract

The present study explores the concept of reenactment of known and unknown war trauma that may be unfamiliar to the readers while also opening up new discourses of understanding and empathy through the lens of Cathy Caruth's trauma theory by focusing on the novel *A Land of Permanent Goodbyes* by Atia Abawi. War fiction, such as Abawi's novel, highlights the concept of psychological trauma as a double wound (known as “outsiders” and unknown as “insiders”) through the textual analysis and characterization of characters by presenting their haunting pasts, present lives, and losses concerning traumatic events. The present study also recounts what the trauma of the unknown means to war-torn survivors by focusing on “trauma as double wounds” from three aspects: cause, effects, and recuperation. Finally, the study concludes with a new paradigm in which trauma is portrayed as a healer rather than a wound.

## Introduction

War and its catastrophic repercussions on the human psyche, which Sigmund Freud termed “traumatic neurosis,” create a “breach” in the “protective barrier” [(1), (p. 29)] and are pervasive in the contemporary world. Before discussing/comprehending war trauma in detail, it is mandatory to shed light on the psychological trauma that arises due to an event/incident. I will stress the limitations of using theory at the psychological level to understand the process of events and war trauma. Now, let us turn to Freud's writing again (deemed necessary for the implication of war trauma), in which he initially focused on the physical trauma related to hysteria, its cause, development, and effects.

Regarding the cause, Sigmund Freud, in his work “*Beyond the Pleasure Principle*,” defined “traumatic neurosis” as being caused by a “factor of freight” [(1), (p. 32)] and being different from hysteria as “a passive sexual experience before puberty” [(1), (p. 152)], usually molestation or seduction by one's father, sibling, or household servant. In this situation, fright mostly comes from the reenactment of traumatic events in unwanted dreams. According to Sigmund Freud, a reenactment of events shows that trauma, unlike anxiety, cannot be achieved or addressed directly by the victim, as it remains unknown and resides in the unconscious but, at the same time, bounces back to the conscious and becomes alive. Moreover, dreams caused by a reenactment of an “event” evoke feelings of horror, fear, shame, and suppression of the unconscious. In such a case, the development of anxiety is characterized by latency or incubation. Freud reiterates that people suffering from mental anxiety are held captive by painful experiences from their distant past. He characterizes the memory of trauma as “a foreign body which, long after its entry, must continue to be regarded as an agent that is still at work” (2), [1893–95], p. 6. The memory desperately clings to their past because it has significant value, and this fixation can last a lifetime.

Consistent with Sigmund Freud's original insights on trauma, another psychologist, Cathy Caruth, in her book *Unclaimed Experience*, introduced the term “trauma theory.” She echoes Sigmund Freud in pointing out that trauma should not be understood as bodily injury but as an injury to the consciousness that, for Jon Allen (3), is a wound and creates “a feeling of utter helplessness (4).” The injury is a result of an emotional shock/traumatic event (war, rape, and social discrimination), which affects the mind more than the body and is “accompanied by fear, helplessness, or horror,” and the “experience of a trauma repeats itself” [(5), (p. 29)], which reappears in dreams and memories. Hence, the trauma of the mind is imprinted in the subconscious as a reality or truth and is unavailable to the consciousness at any given moment, which Cathy Caruth called “the unknown.” She further argues that the victim does not understand the nature of the traumatic event.

“War trauma,” as defined by Caruth (5) (1) war survivors also suffered from and experienced the trauma of known and unknown events), is recognized as “traumatic neurosis,” (2) which manifests as an unwilling reenactment of an event, which Sigmund Freud called “repetition compulsion,” which is not a “memory but [act] as an action” [(1), (p. 150)]. Since World War I, the concepts of “shell shock,” “war neurosis,” or “war trauma” have become common terms due to the development of traumatic neurosis analysis. [(6), (p. 241)] considers the neurological harm caused by bombing and the cruelty of war an “unconscious conflict,” hence the phrase “shell shock.”

Caruth expanded on Freud's concept of traumatic neurosis, arguing that the effect of a painful experience, such as the dropping of a shell/bomb, can be found in the mind through microscopic changes or changes in the central neural system, which is also called traumatic neurosis of war [(5), (p. 44)]. However, the traumatic neurosis of war indicates a great internal unsolvable dispute, which Caruth viewed as the “repetition of a traumatic event which remains unavailable to consciousness but intrudes repeatedly on sight” (5). As a result, the experience of a traumatic event wreaks havoc on and causes problems for the victim's life, as observed by Brown (7), who said that when a war lasts more than 5 weeks, around 8% of those affected people become victims of neural stress (595). Therefore, regarding the brutality of wartime violence, trauma and its effects on the psyche cause mood disorders and PTSD (post-traumatic stress disorder). According to a study conducted by the University of Oxford's Department of Psychiatry, the most commonly diagnosed mental health disorders for refugees (from ethnic groups) in Western countries are anxiety, depression, and PTSD. Further, Scheuermann claimed that, as *The Guardian* notes, “a report last year (i.e., 2016) by the German Federal Chamber of Psychotherapists said that 40–50 percent of people arriving in Germany suffered from Post-Traumatic Stress Disorder (PTSD, with half also suffering from depression” (n.p.).

In addition to psychological wounds, Caruth's and Freud's writings are also about the voice/narration that describes the stories behind those (known/unknown) wounds, and that, for Caruth, is “a voice that is paradoxically released through the wounds” [(5), (p. 2)]. When dealing with literary texts, narrating traumatic stories offers two perspectives on painful events. First, texts show what is recognized (known trauma) during the first meeting with a terrible event, i.e., an actual incident that happened in the life of the victim, which called “witness to oneself” (8). Second, texts show the absent part as “the unknown” and “not locatable” [(5), (p. 4)], which appeared through discussion/narration. Caruth further developed her idea of narration concerning literary texts and said that “if flashbacks of the traumatized persons thus engage Sigmund Freud's interest, it is because they bear witness for survival that exceeds the very claims and consciousness of the one who endures it” (5). Hence, for Caruth, Freud used literature to describe the painful experience because literary texts (like psychological analysis) are used to know and not know unavailable truths and realities.

According to Caruth, the traumatized victim recovers from trauma by repeatedly recalling or going through past events. D. Laub pointed out that “the survivors [of the war] did not only need to survive so that they could tell their story but also needed to tell their story to survive” (8). Thus, articulation is a source of catharsis for victims. The main idea behind this ideology of catharsis is latency: “the historical power of the trauma is not just that the experience is repeated after it has been forgotten, but that it is only in and through its inherent forgetting that it is first experienced at all” [(5), (p. 17)]. This concept of latency implies a deferral to perception and the assimilation of occasions into the mind's memory.

Furthermore, the latency of experience is also responsible for redundancy, the second significant idea attached to Cathy Caruth's concept of trauma injury. This insight hypothesis is the emergence of hope in the stories of destruction. Trauma not only destroys lives but also serves as a healer to restore the victim to a normal existence (9).

Besides psychiatrists, writers like “Wilfred Owen” focused on psychological trauma to find a cultural definition of trauma that is as useful as the implications of trauma theory for determining the problems. Similarly, Sarah Cole also pointed out that the writers “emblematized the war not in death or in physical injure [sic] but in the psychological wound” (Cole, 194). Likewise, Galsworthy argues the following:

Stress has a more powerful and deep influence over literature as compared to war itself. In literary fiction, a common man or protagonist becomes a symbol and sign of psychological crisis who undergoes traumatic problems (10).

## Literature review

Cilano (11) examines Pakistani literature to determine how the country became entangled in the US war on terrorism. Muslim migrants' identity issues and immigration in the context of 9/11 are major issues in these studies, as the protagonist Changez in the novel *The Reluctant Fundamentalist* experiences. However, Arcimaviciene and Baglama (12) and Shamsie (13) referred to the work as “topical and prescient,” highlighting the fundamental social and political challenges as well as an individualistic battle where nativists want to make refugees “modern-day janissaries” by assimilating, that Collier (14) calls and considers as “hunkering down,” and treating them as others, which Elahi (15) illustrates that Muslims have “alien customs” for nativists. Such dichotomous behavior by nativists due to cultural clashes has a negative effect on the identity of Muslim migrants and causes cultural trauma.

Looking critically at the overall body of literary works such as Waheed Mirza's *The Book of Gold Leaves* (16), Basharat's *Curfewed Nights* (17), and Mohsin Hamid's *Exit West* (18) *, as well as* Khalid Hosseini's *Kite Runner* (19) and *Sea Prayers* (20) and Dave Eggers' *What is the What* (21), we observed the effect of the war atrocities on people's lives. However, *House Made of Dawn* (22) by N. Scott Momaday discusses the effects of the war and its endurance, which led Native Americans to suffer from cognitive “disabilities.” Bhabha (23) relates such disabilities with “unhomeliness” and “historical migration” (p. 141). Regarding disabilities and collective identity, psychopathologist van der Kolk et al. (24) argues that people with less support or attachment to social values during exile undergo severe traumatic reactions and may negatively influence “health and psychopathology.” However, Alexander (25) describes it in a different light and calls trauma a wound that is “not something naturally existing; it is something constructed by society and from events themselves” (p. 2, 3).

While paying attention to the relocation of migrants, researchers focused on humanitarian crises, economic instability, social issues, and political problems but missed the subject of psychological trauma. For example, Fisher (4) said that refugees need urgent help and safe havens, but nativists prioritize border security over fundamental human rights and treat migrants as second-class citizens. In contrast, Ahmad Mir (26) examined the migrants' concerns about individual rights and shelters and called the notion of nationality into question. Sadiq et al. (27) highlighted the social, political, and economic upheaval as the reason for migration and analyzed Hamid's *Exit West* (2017) as a current portrayal of the forced movement of people around the globe from places experiencing anarchy. Amanda Lagji's (28) research added a postcolonial perspective to the study of the mobility of migrants. Similarly, Gheorghiu (29) used magical realism to investigate the refugee issue emotionally and politically.

Moreover, this paper on the fiction *A Land of Permanent Goodbyes* in the context of war problems and humanitarian crises has been found to have significantly contributed to an already existing body of knowledge regarding the issues of racism, ethnocentrism, economic instability, and social and political problems. Researchers viewed these texts through multiple lenses. Nevertheless, despite the rise in painful war events and their effects, the war trauma from a psychological (known and unknown) perspective did not get the attention it deserved. However, this study focuses on how relocation during a war leads to war trauma, causing identity crises and affecting victims' lives. The novel *A Land of Permanent Goodbyes* (2018) by Atia Abawi deals with loss, rape syndrome, the “othering” of migrants, and their struggle with identity, alienation, and survival. To the best of my knowledge, no researcher has yet analyzed the selected novel to highlight war trauma by applying the psychoanalytical approach of Cathy Caruth's trauma theory.

## Methodology and theoretical concept

The study drew its methodology from Cathy Caruth's trauma theory, retained the memory of “unwitting” traumatic events, and focused on the notion of “the unknown” and “not locatable” trauma [(5), (p. 4)], its context, and the selected novel. Caruth [(5), (p. 11)] defined trauma as an experience of “catastrophic events in which the response to the event occurs in the often delayed, uncontrolled, repetitive appearance of hallucinations and other intrusive phenomena” (23). The concept of “unknown” trauma refers to/occurs at an individual level and can also be connected with Sigmund Freud's terms “traumatic neurosis” and “factor of freight” (10). In this situation, fright mostly comes from the reenactment of traumatic events that the patient “reproduces not as a memory but as an action; he repeats it without knowing that he is repeating” [(1), (p. 10)], in unwanted dreams that remain unknown and reside in the unconscious but later bounce back to the conscious and become alive.

Keeping in view these definitions of psychological trauma concerning traumatic events, the study examines war trauma and identity issues of refugees in Atia Abawi's selected novel *A Land of Permanent Goodbyes*, which was written after Syrian and before the Russian attack on Ukraine. By doing so, the researcher used the “text and context of an event” [(30), (p. 34–5)] to examine its treatment and determine the causes of psychological trauma.

Cathy Caruth posited that, in response to Sigmund Freud's concept of trauma and dream, a victim does not fully experience the trauma immediately because it is “too soon, too unexpectedly, to be fully unknown” [(5), (p. 142)]; instead, the victim repeats the events, carrying with them the struggle to come to terms with the experience. Caruth viewed trauma as the “repetition of a traumatic event, which remains unavailable to consciousness but repeatedly intrudes on sight, suggesting a larger relation to the event that extends beyond what can simply be seen or what can be known” (5). Here, Caruth's basic diagnosis of disastrous trauma incidents does not include the complication of “unassimilated nature,” which is unknown in the beginning but “gets back to haunt the victim of trauma later on” [(5), (p. 4)]. The unknown or “apparently unharmed” [(5), (p. 18)] part of the painful incident distracts the survivor from expressing trauma and makes him familiar with known trauma through the repetition of recurring thoughts, which Ann Wolbert Burgess and Lynda Lytle Holmstrom call “the traumatic neuroses of war” (61). Because the initial shock is buried in memory, it is frequently impossible to cognitively grasp the trauma, even if remnants of it surface in the form of flashbacks and hallucinations. It constantly teases the victim and causes many cognitive changes, which Freud described as the following:

“one must suppose rather than the physical stress and trauma-or more specifically the remembrance and memory of traumatic incident-acts like an unknown element which after a long time of the actual happening continues to be termed as an element that is still very active” [(1), (p. 06)].

By extending the idea of known and unknown trauma with respect to war events, a clinical and forensic psychologist investigated and based her study on Maria P. P. Root's concept of “insidious trauma,” which maintains that “traumatic effects of oppression that are not necessarily overtly violent or threatening to bodily well-being at the given moment, but that do violence to the soul and spirit” [(31), (p. 107)]. Here, insidious trauma qualifies and supports Caruth's concept that trauma is hidden but alive. Hence, trauma is a silence that kills or haunts the victim continuously, and the painful experiences are unable to be fully described by the victims. The victim desires to end his life and feels like a dead person, in which death becomes a strong driver that represents the victim's inner fear and insecurity [(32), (p. 211)].

According to Caruth, a traumatic event causes a double wound, where a victim with a painful experience faces trauma twice: once as the witness of his trauma and again in the reexperiencing of the traumatic event [(5), (p. 89)]. Another psychologist, D. Laub, contributed to the idea of witnessing Caruth by pointing out that it exists on three levels. He asserted that “witness to oneself” (8) is the first level of witnessing and experiencing the event directly. In contrast, the second level of witnessing requires the testimony of interviewers. The third level of witnessing involves the testimony process, in which the witnessing can either be live or experienced *via* video or audio recording. These three levels of witnessing are important to consider if one is to grasp the true nature of trauma. Likewise, the survivor of traumatic events also undergoes dual trauma, i.e., the painful experience of firsthand witnessing death (known) and the trauma of surviving death (unknown) and the subsequent anxiety, depression, and nightmares, which explain the “real story” that [(33), (p. 3)] calls “episodic” memory as opposed to the more general “semantic” memory. Caruth further said that those who have witnessed horrific violence or attacks could not forget these “indelible” memories. Such hyper-visual trauma cannot be altered in the unconscious and will hurt the victim.

In relation to the experience of a traumatic event and witnessing it, Caruth focused on the traumatic effect on a traumatized person by calling it a “threat [that] is recognized by a mind one moment too late” [(5), (p. 64)] with an intrusion of “nightmares” or “experiences in flashbacks” [(5), (p. 60, 65)] that disrupts the victims' regulated and balanced pattern of the world, as was the case for survivors of the Vietnam War or concentration camps. In this respect, Abram Kardiner, a psychiatrist, observes victim's reactions toward unknown/unwanted events by “employing language suggestive of his trying to defend himself during a military assault” [(34), (p. 58)], where victims' fixation can last a lifetime [(1), (p. 12)] because victims receive “a shock of a contingent encounter” in terms of flashbacks or nightdreams that is like “a grain of sand” which “ruins the balance of the symbolic universe of the subject” [(35), (p. 171)] and are held captive to painful experiences of their distant past.

Caruth (like Freud) merged the language of literature to illustrate the concept of psychological trauma that goes toward the fact between known and unknown trauma relations. Language and reading trauma theories are integral. Language/reading preserves the trauma through literary stories. The victim always desires to speak about the traumatic experiences and losses that Freud calls “bearing witness” (5); hence, literary fiction becomes a source of narration to highlight those traumatic experiences. For instance, a witness to the Holocaust can relive and experience a state of trauma that has been repressed, but traumatic experiences are so overwhelming that he cannot describe them accurately and clearly. As D. Laub points out, “the survivors [of the war] did not only need to survive so that they could tell their story but also needed to tell their story to survive” (8). Sometimes, articulation becomes a source of catharsis for victims in the sense that sharing feelings and explaining the scars and injury serves as an outlet, which is not possible in other ways; however, the victim loses the chance to explain trauma accurately. Traumatized people need sufficient time and a specific context to address and articulate the traumatic events. This ambiguous situation can lead to psychological problems for the affected person and the whole community.

Michel Foucault emphasizes that, keeping in mind the views surrounding trauma, “power is everywhere” [(36), (p. 8)]. Thus, the trauma representation of fiction through the power of narration plays an important role in describing historical events based on the facts of a country and society. The main reason for choosing “*A Land of Permanent Goodbyes*” was that this study aimed to highlight the effects of the war on migrants by relying heavily on Cathy Caruth's trauma theory.

## Discussion and analysis

### War trauma: Cause and double wound

*A Land of Permanent Goodbyes* traces the life of a young refugee, Tareq, from a war-torn country, who loses most of his family members to the war and is forced to flee but suffers from what [(37), (p. 465)] call shell shock, causing long-term negative effects such as nightmares, anxiety, and depression. These effects bring about a massive change in the psychological organization of the person before and during exile, creating an atmosphere for psychological trauma.

According to Cathy Caruth's definition of trauma, it is “too soon, too unexpectedly, to be fully unknown events” at the time of the event [(5), (p. 142)], although it returns later. Caruth's definition of trauma and loss qualifies the analysis of the protagonist, Tareq, who leaves his “loved ones without time to mourn” (210) to keep himself safe for the survival of the fittest. His act, according to Caruth, is “apparently unharmed, from the spot where he has suffered a shocking accident,” but he experiences a series of psychical symptoms, for instance, night dreams ascribed to traumatic neurosis. Moreover, Caruth's interpretation of “unknown” and “not locatable” trauma [(5), (p. 4)] shows Tareq's experiences of loss, where he “dreamed of [his native] home” in the host country and considers that “it is not [his native] home” [(38), (p. 100)] and that for Warsan Shire, “it is the mouth of a shark” (*Home*, n.p.) for every migrant. Similarly, a critic, Edward Said (39), comments that the loss of a home is an “unbearable rift forced between the self and its true home” (174), which, in Caruth's words, is not fully known until the victim, Tareq, has a “nightmare” and feels that it is “from trauma in his sleep” [(38), (p. 11)]. Hence, the victim of a traumatic event (for war-torn refugees like Tareq) suffers from double pain/double wound. The horror of leaving their homeland and loved ones is a crisis of death (known), but the lingering fear of losing their own lives in the conflict is a crisis of life (unknown) that drove them to leave their homeland and loved ones before the battle devoured them all. Therefore, trauma has an unknown characteristic that returns to the victim's consciousness and brings the wounds to life, haunting them intermittently.

Sigmund Freud on the concept of loss and double wounds (like Caruth) in his seminal work *Beyond the Pleasure Principle* also suggests that repetitive behavior, flashbacks, and intrusive thoughts are imprinted in the subconscious as a reality or truth and are unavailable that Cathy Caruth (5) in her book *Unclaimed Experience* calls “never be fully known” (31) to the conscious mind immediately but return later. For instance, like Tariq, his father sees nightmares and “the ghosts of his other children and wife [who] constrict his every breath” [(38), (p. 31)] after deciding to relocate his family out of the war-torn city. Hence, Freud's and Caruth's ideas reveal that trauma is created through unprecedented events where a victim cannot understand the nature of the traumatic event, which is so strong that it disturbs awareness of the world and later reappears in dreams and memories due to the “loss of an object that has been the libidinal-object [...] such as a loved one, one's country, or liberty” [(1), (p. 243)]. Freud mentioned bad dreams and nightmares in his book *On Dreams*, which Mohsin Hamid also depicted in his book *Exit West*. In agreement with Freud and Caruth, (40) pluralistic trauma model deals with a traumatic event, the self-identity of victims, and its effects on hallucinations and nightmares. As Niederland (41) explains, many people who survive horrendous attacks and suffer from severe trauma feel they are “different people” afterward.

### Double wounds' effects and symptoms

In addition to losing a home, the war severely affects a victim's identity. Caruth calls it a “horrendous experience,” which is possible to access in normal consciousness later. In this study, “normal consciousness” refers to the trauma that resides in the unconscious in a timeless, speechless state that causes continuous pain and damage to the psyche [(5), (p. 160–63)]. Caruth's interpretation proves true as the effects of the war gradually appear later and make Tareq suffer from hallucinations. He starts to see his dead brother “wearing the same clothes from the last day he saw him” and considers himself “crazy” [(38), (p. 139)].

Similarly, Tariq's other brother “screamed in the night” when he slept while Ahmed (a 25-year-old doctor) felt like an “already dead” person when his “future came to an end” [(38), (p. 22)] like many other migrants who are “forced to fight” [(38), (p. 37)] for something they do not believe in. The feeling of being a “crazy and dead” person speaks volumes and shows a sensation of “any excitations” [(1), (p. 29)] in the unconscious that disrupts the victim's regulated and balanced pattern of the world, which Sigmund Freud called a “protective shield” (42), ensuring that it shatters the balance of the psyche and the normal life of Tareq, his brother Salim, Ahmad, and many others like them. Hence, the change of identity as a result of excitation/unknown trauma arises from the unconscious, which according to the *Substance Abuse and Mental Health Services Administration*, “affects the individual's physical, social, emotional, or spiritual wellbeing” [(42), (p. 2)] and weakens the normal emotional and subjective reactions to life and brings enduring mental disturbances for the victims who secure from war. Sigmund Freud's view on the life of survival was that, as the brain is protected by a film/membrane that plays a pivotal role in stimulus control, if a boost goes through the layer or the membrane, it inadvertently influences the psyche. Sigmund Freud used that method to relate trauma to its substantial injuries, presenting it as an injury to the defensive shield of the mind. We describe trauma as any shock from the outer world that a mind cannot bear, which results in a breakup of the protective shield of the mind.

Awabi's work also depicts neurosis effects on females concerning sexual exploitation and bodily injury during wartime, which works as a rule for “the development of a neurosis” [(1), (p. 12)], but its effects appear on the psyche like a waking memory in the mode of the dream as a symptom. Tareq comes across news about Muzhgan, another migrant teenage girl, that “her smuggler in Turkey had repeatedly raped her before he finally set her free […] and she “was terrified every day,” hence she “will never be the same” [(38), (p. 256, 257, 271)], which for Guglielmo [(41), (p. 421)] is a “different person” because the victim would not be able to get rid of traumatic dreams and nightmares. The traumatic experience of Muzhgan makes her numb and silent and links her with language loss because the trauma of losing her virginity is not simply a wound of the body that can heal with time (5); rather, it occurs unexpectedly and is known to the victim. Similarly, Dominick (43) also talks about trauma victims through his theory of acting out and working through to show the response of those who have witnessed all the horror and survived. He puts forth that “a person is disturbed or occupied by the past and is forcibly caught in a forced repetition of traumatic scenes,” adding that in this case, “tensions broke out, and it was as if one had returned and repeated a sad scene” [Dominick (43), p. 21].

Caruth commented on Sigmund Freud's On Dream Theory, saying that Freud compared unknown trauma to a recurring nightmare during sleep that produced a fear-based physical response, even after waking up: dreams that involve memories of traumatic experiences return patients to the psychological state induced by accident, a circumstance from which they awaken in distress (61). Moreover, on “*Rape Trauma Syndrome*” of war-torn migrants, a study showed that “the terrifying flashbacks and nightmares” cause traumatic “neuroses of war” among victims (Ann Wolbert Burgess and Lynda Lytle Holmstrom, 61). In such situations, dreamers, like Muzhgan and Tareq, encounter a person who is struggling for life, and in the dream state, that person exists as if in reality, whether still living or deceased.

### Recuperation: Double wounds as a healer

Cathy's model's definition shows that once the “unknown” trauma is “spoken” and comes to consciousness, it becomes less “traumatic,” which, for Freud, was a “perplexing experience of survival”; otherwise, the victim remains in the “black hole” of trauma [(5), (p. 9–11, 62)]. In regard to Caruth's definition, Tareq regains a foothold and re-establishes his identity in his host country (unlike other migrants and family members who do not speak) by narrating the exact and meaningful message of his traumatic experience and thus finds the resilience and courage to end his harrowing journey of unknown trauma in Greece. Although trauma can be repeated in the lives of survivors by narrating it, no traumatic event repeats itself with the same intensity. As Gayatri Chakravorty Spivak (44) stated, “What is said to return is not the repressed but a version of it; the repressed is not the thing that we return intact” (67). The return intact also interferes with (as is the case of Tareq) and stops the cycle of permanent traumatic shock, the psychic wounds after narrating and going through it. However, this is not merely a repetition and return intact of unknown trauma but rather “a controlled, explicit, critically controlled process of repetition that significantly changes a life by making possible the selective retrieval and modified enactment of actualized past possibilities” [Dominick (43), p. 14]. Therefore, if strictly enforced, repeated speech creates a sense of agency for those whose mental lives dominate the rules of others.

Similarly, LaCarpa, in his work *Writing History, Writing Trauma* (2001), talks about two states with reference to trauma, i.e., acting out and working through. Acting out suggests the state in which the person is traumatized, engulfed in his fears of the past, and unable to escape that situation. He relives the same traumatic experience again and again and is unable to come out of that fear, while in the case of working through, the victim can differentiate between moments of trauma that he experienced in the past and moments of everyday life.

## Conclusion

Involving trauma survivors in a dialectical process of listening and answering to the right to vote, empowering them within the context of a sympathetic denomination, is essential to normalizing their state or helping them recover from trauma. The first step to achieving this goal is trauma rehabilitation. The rehabilitation of trauma exposure supports the victim in working through the damaging force of the shock, which is repeatedly implicated in reviving their suffering, which exacerbates over time. Narrating a traumatic event through talk helps traumatized people work through the destructive force of trauma. This process enables them to remove traumatic highlights of the event repeatedly.

In addition to a dialectical procedure, the enactment of trauma may also help the victim recover from trauma as the reenactment process is based on the quality, purpose, condition, and nature of reenactment within the social context in which it occurs. The social context covers the social position of the traumatized person and creates empathy for the witness or audience for the victim. The deliberate recovery of the trauma of an ongoing sympathetic social environment helps to alleviate unbearable stress or at least completely separate the victims from it. It not only harmonizes the survivors' trauma and traumatic events (such as war), which shock and embarrass their victims, but also establishes an emphatic link between society and the victim.

## Data availability statement

The original contributions presented in the study are included in the article/supplementary material, further inquiries can be directed to the corresponding author.

## Author contributions

All authors listed have made a substantial, direct, and intellectual contribution to the work and approved it for publication.

## References

[1] Freud S. Beyond the Pleasure Principle, trans. James strachey the standard edition of the complete psychological works of sigmund. Freud. (1961) 18:1920–2.

[2] Breuer J. The standard edition of the complete psychological works of sigmund freud. In: Translated from the German Under the General, Editorship of James Strachey in Collaboration with Anna Freud Assisted by Alix Strachey and Alan Tyson. London: The Hogarth Press, The Institute of psycho-analysis. (1955) p. 1893–5.

[3] Allen JG. Coping with Trauma: A Guide to Self-Understanding. Washington, DC: In Amer Psychiatric Publication (1995).

[4] Fisher BL. Doors to safety: exit west, refugee resettlement, and the right to asylum. Mich L Rev. (2018) 117:1119. 10.36644/mlr.117.6.doors

[5] Caruth C. Unclaimed Experience Trauma, Narrative, and History. Baltimore, MD: The Johns Hopkins Press Ltd. (1996).

[6] Szykierski D. The traumatic roots of containment: the evolution of Bion's metapsychology. Psychoanalytic Q. (2010) 79:935–68. 10.1002/j.2167-4086.2010.tb00472.x 21141783

[7] Brown ML. Screams Somehow Echoing: Trauma and Testimony in Anglophone African Literature. University of Maryland Libraries (2008).

[8] Laub D Podell D. Art and trauma. Int J Psychoanal. (1995) 76:991–1005. 8926145

[9] Caruth C. Explorations in Memory. Baltimore, MD; London (1995). p. 2012–13.

[10] Mir Meha. Assimilation Healing of War Trauma: A Study of Exit West. (2021). Available online at: https://www.oeconomiacopernicana.pl

[11] Cilano C. Contemporary Pakistani fiction in English: Idea, Nation, State. London: Routledge (2013).

[12] Arcimaviciene L. Migration metaphor and myth in media representations: The ideological dichotomy of “them” and “us”. Sage Open. (2018) 8:2158244018768657. 10.1177/2158244018768657

[13] Shamsie M. How the West Was One. (2017). London: Newsweek Pakistan. Available online at: https://www.newsweekpakistan.com/how-the-west-was-one/ (accessed December 1, 2022).

[14] Collier P. Exodus: How Migration is Changing Our World. Oxford University Press (2013).

[15] Elahi FOK. The Runnymede Trust | Islamophobia: Still a Challenge for Us All. London: Runneymede (2017).

[16] Rankine D. The Book of Gold. Penguin. (2003). Available online at: https://www.penguin.co.uk/books/253366/the-book-of-gold-leaves-by-waheed-mirza/9780241970829 (accessed December 1, 2022).

[17] Peer B. Curfewed Night. New York, NY: Random House India (2009).

[18] Hamid M. Exit West. New York, NY: Riverhead Books (2017).

[19] Hosseini K. The Kite Runner. New York, NY: Riverhead Books (2013).

[20] Hosseini K. Sea Prayer. New York, NY: Riverhead Books (2018).

[21] Eggers D. What is the what : the Autobiography of Valentino Achak Deng : A novel. New York, NY: Vintage publisher (2007).

[22] Momaday NS. House Made of Dawn. New York, NY: Harper Perennial Modern Classics (1999).

[23] Bhabha HK. The Location of Culture. 1994. Reprint. New York, NY: Routledge Classics (2004).

[24] van der Kolk BA McFarlane AC. Traumatic Stress: The Effects of Overwhelming Experience on Mind, Body, and Society. New York, NY: Guilford Publications (2012).

[25] Alexander JC. Trauma: A Social Theory. John Wiley and Sons (2013).

[26] Ahmad Mir M. Global refugee crisis: a study of mohsin hamid”s novel exit west. Int J Interdis Res Arts Human. (2018) 3:15–6. 10.5281/zenodo.1135244

[27] Sadiq N Saleem AU Javaid S. Subjectivity, power affairs and migration: a foucauldian analysis of hamid's exit west. Glob Regional Rev. (2020) 1:584–93. 10.31703/grr.2020(V-I).61

[28] Lagji A. Waiting in motion: mapping postcolonial fiction, new mobilities, and migration through Mohsin Hamid's Exit West. Mobilities. (2018) 14:218–32. 10.1080/17450101.2018.1533684

[29] Gheorghiu OC. As if by magical realism: a refugee crisis in fiction. Cult Intertexts. (2018) 8:80–93. 10.35219/cultural-intertexts.2018.05

[30] Jeffrey CA Eyerman R Giesen B Smelser NJ Sztompka P. Cultural Trauma and Collective Identity. University of California Press (2004).

[31] Brown LS. Not outside the range: one feminist perspective on psychic trauma. Am Imago. (1991) 119–33.

[32] Herman JL. Trauma and Recovery. New York, NY: Basic Harper (1992).

[33] Hwangbo K. Trauma, Narrative, and the Marginal Self in Selected Contemporary American Novels. University of Florida (2004).

[34] Kardiner A. The Traumatic Neuroses of War. New York, NY: Paul B Hoeber Inc. (1941).

[35] Žižek S. The Sublime Object of Ideology. New York, NY: Verso Books (1989).

[36] Culler J. Identity, Identification, and the Subject. Literary Theory. A Very Short Introduction. Valerie: Oxford University Press (2000). p. 110–41.

[37] Laplanche J. The Language of Psychoanalysis. D. Nicholson-Smith, editors. London: Norton (1974).

[38] Abawi A. A Land of Permanent Goodbyes. Philomel (2018).

[39] Said E. Reflections on Exile. Said EW Reflections on Exile and Other Essays. Cambridge, MA: Harvard University Press. (2000) p. 174.

[40] Balaev M. The Nature of Trauma in American Novels. Northwestern University Press (2012).

[41] Niederland WG. The survivor syndrome: further observations and dimensions. J Am Psychoanal Assoc. (1981) 29:413–25. 10.1177/000306518102900207 7264182

[42] Abuse S. Mental health services administration, trauma and justice strategic initiative. In: SAMHSA's Working Definition of Trauma and Guidance for Trauma-Informed Approach. Rockville, MD: Substance Abuse and Mental Health Services Administration (2012).

[43] Dominick L. Writing History, Writing trauma. Baltimore, MD: Johns Hopkins UP (2001).

[44] Spivak GC. Outside in the Teaching Machine. New York, NY: Routledge. (2012).

